# Supplementary material for: A homozygous loss-of-function mutation leading to CYBC1 deficiency causes chronic granulomatous disease
Source: Nat Commun. 2018 Oct 25;9:4447. doi: 10.1038/s41467-018-06964-x (PMC6202333; doi:10.1038/s41467-018-06964-x)
Supplement: Supplementary file 1 — Supplementary Information [file 41467_2018_6964_MOESM1_ESM.pdf]

Supplementary Information

A homozygous loss-of-function mutation leading to CYBC1 deficiency causes chronic granulomatous disease

Arnadottir et al.

Supplementary Note 1: Detailed clinical description for the two probands.
Supplementary Note 2: Variants detected in the two probands.

Supplementary Figure 1. CYBC1 mRNA expression in white blood cells of heterozygous carriers of *CYBC1* p.Tyr2Ter (*n*=29) and non-carriers (*n*=2,430).
Supplementary Figure 2. CYBC1 mRNA allele specific expression in white blood cells of heterozygous carriers of *CYBC1* p.Tyr2Ter (*n*=29).
Supplementary Figure 3. CYBC1 protein expression in lymphocytes from *CYBC1* p.Tyr2Ter homozygous individuals D and F and their matched (age and sex) non-carriers.
Supplementary Figure 4. CYBC1 protein expression in lymphocytes from four heterozygous carriers of *CYBC1* p.Tyr2Ter and a matched (age and sex) non-carrier for each.
Supplementary Figure 5. gp91^{phox} protein expression in monocyte-derived macrophages from *CYBC1* p.Tyr2Ter homozygotes and their matched (age and sex) non-carriers.
Supplementary Figure 6. Pedigrees of the six additional p.Tyr2Ter homozygotes (individuals C-H).
Supplementary Figure 7. Gating strategy for analysis of Rhodamine 123 fluorescence in neutrophils.
Supplementary Figure 8. Uncropped western blot accompanying Fig. 2d.
Supplementary Figure 9. Uncropped western blots accompanying Supplementary Figs. 3, 4, and 5.

Supplementary Table 1. Average coverage of the six genes in which mutations are known to cause CGD, including *CYBC1*.
Supplementary Table 2. Phenotypes of Icelandic individuals homozygous for NP_000092.2:p.Arg90Trp in *CYBA*.
Supplementary Table 3. Association of *CYBC1* p.Tyr2Ter (rs778180128) with IBD and height in Iceland.
Supplementary Table 4. Height information on the eight individuals homozygous for *CYBC1* p.Tyr2Ter.
Supplementary Table 5. Homozygous coding variants in the canonical transcript (NM_001033046.3 / NP_001028218.1) of *CYBC1* in Iceland[1].

Supplementary References.

Supplementary Notes

Supplementary Note 1: Detailed clinical description for the two probands.

The two brothers (individuals A and B) were born in 1991 and 1993. Their parents are unrelated, they have two healthy older brothers, and a younger sister with hearing impairment of unknown origin. At 7 and 9 years of age, the brothers developed severe diarrhea and were diagnosed with Crohn's disease. Repeated biopsies from bowel confirmed granulomatous lesions, consistent with Crohn's disease. However, therapy for Crohn's disease, including steroids, was unsuccessful. The younger brother was at one time diagnosed with a *Legionella* infection, successfully treated, and also developed an ulcer in his mouth that tested positive for the bacteria *Burkholderia cepacia*. This bacteria rarely causes infections except in immunocompromised patients, including patients with chronic granulomatous disease (CGD)[2].

CGD commonly presents with gastrointestinal symptoms and CGD gastrointestinal granulomas can be identical to Crohn's granulomas[2]. The younger brother also had frequent ulcers around his nostrils, consistent with CGD, that were at the time thought to be part of Crohn's. Typically, CGD patients would be expected to have more frequent infections than observed for the two brothers[2]. It could therefore be argued that the brothers had a somewhat milder, although severe, form of CGD.

Following the initial indications of CGD, including infection caused by *Burkholderia cepacia*, several tests were performed through the University Hospital of Iceland. Typical immunological tests showed negative results. At the same time (in 2008) a phagotest, for phagocytosis, and burst test (neutrophil oxidative burst test by dihydrorhodamine (DHR) 123 assay), were performed. The tests revealed a severely impaired neutrophil oxidative burst, for both brothers (see Fig. 1b). The tests were repeated with the same results and blood samples were subsequently sent to the Netherlands where the results from Iceland were confirmed.

In the following months, it became increasingly difficult to treat the brothers for gastrointestinal symptoms. A treatment of repeated periods with steroids showed only limited and temporary improvement. The condition of older brother, who also developed an enlarged liver and spleen, was particularly severe. Due to the progression of their disease, it was decided that the brothers would undergo hematopoietic stem cell transplantation (HSCT) in Newcastle, both from unrelated donors. The older brother developed severe complications after receiving the transplant, and died in 2009. The younger brother was successfully transplanted in 2010, and has been symptom free for eight years (in 2018). All in all, the brothers had clinical symptoms well compatible with CGD, as well as biological testing supporting the clinical diagnosis of CGD (both in Iceland, repeatedly, and in the Netherlands).

Supplementary Note 2: Variants detected in the two probands.

The two probands share two rare (as defined in Methods) coding genotypes that are absent from their unaffected parents. Both genotypes consist of homozygous variants: a missense variant in *GCGR* (NM_000160.3:c.449G>A; NP_000151.1:p.Ser150Asn; hg38 position chr17:81,811,277; MAF=0.33%) and the stop-gained variant in *CYBC1* (NM_001033046.3:c.6C>G; hg38 position chr17:82,449,249; MAF=0.76%) described in detail in this study. The missense variant in *GCGR*, NP_000151.1:p.Ser150Asn, is homozygous in three other Icelanders. *GCGR* encodes a glucagon receptor, involved in the regulation of blood glucose levels and glucose homeostasis. A single missense variant in *GCGR*, NP_000151.1:p.Gly40Ser, has been reported to associate with type 2 diabetes. No other phenotypic traits have been linked to homozygosity of variants in *GCGR* in humans. Homozygous knockout mice display abnormal glucagon levels with other phenotypes secondary to abnormal glucagon levels[3].

Supplementary Figures

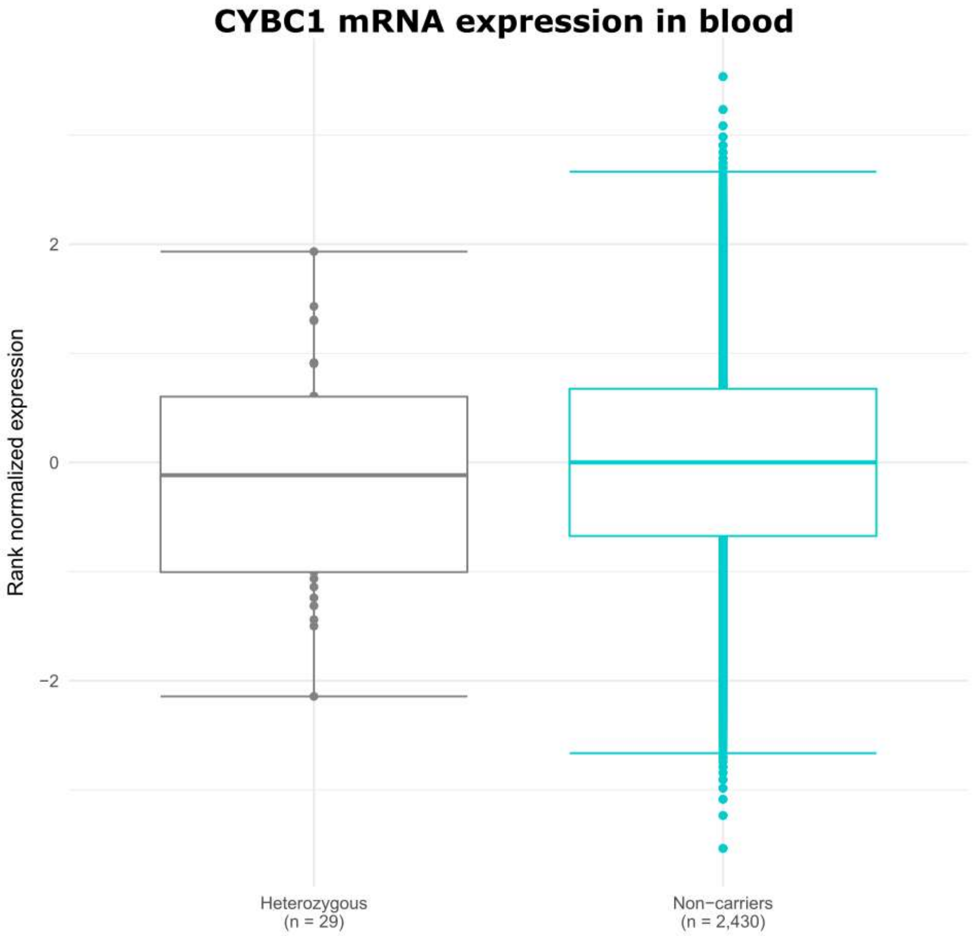

Supplementary Figure 1. CYBC1 mRNA expression in white blood cells of heterozygous carriers of *CYBC1* p.Tyr2Ter (*n*=29) and non-carriers (*n*=2,430). There is no indication of reduced mRNA expression in heterozygous carriers versus non-carriers (*P* = 0.86; Effect (95% CI) = 0.03 SD (-0.36, 0.30); likelihood ratio test).

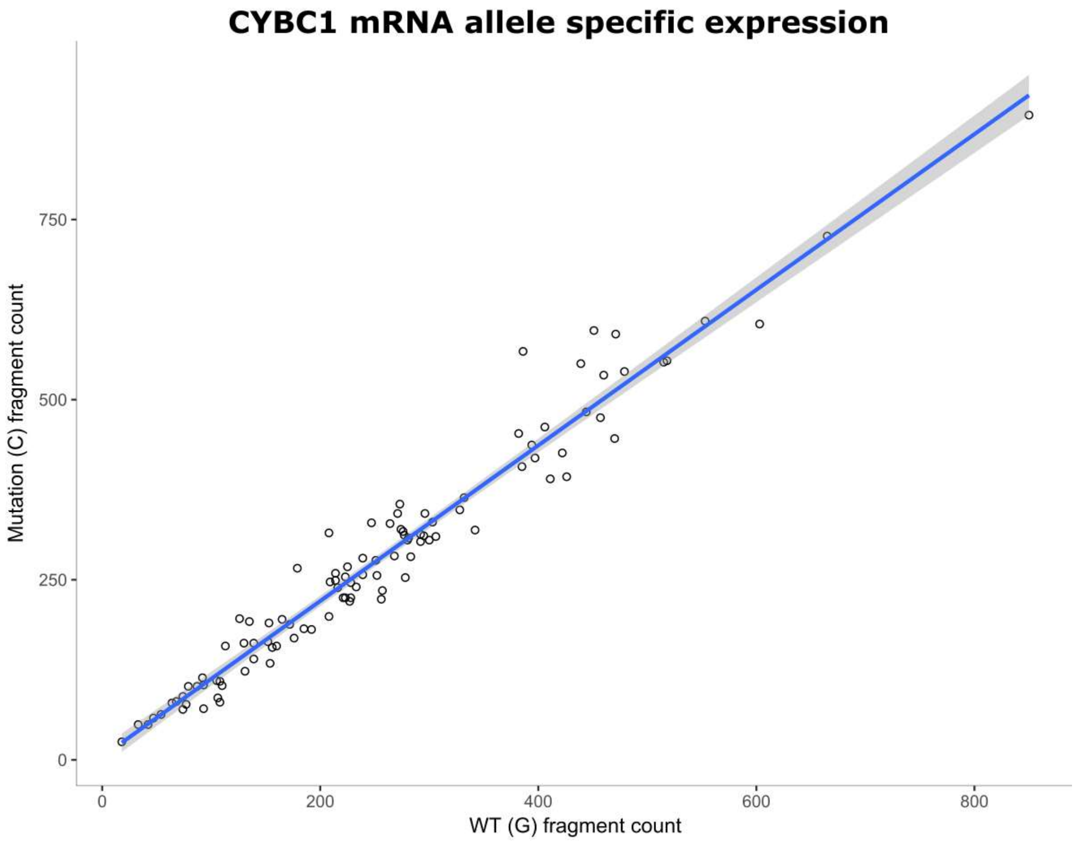

Supplementary Figure 2. CYBC1 mRNA allele specific expression in white blood cells of heterozygous carriers of *CYBC1* p.Tyr2Ter (*n*=29). Shown are mRNA reads with mutation (C) versus reads with the wild-type allele (G). C counts are 377±158 (51%) and G counts 360±156 (49%).

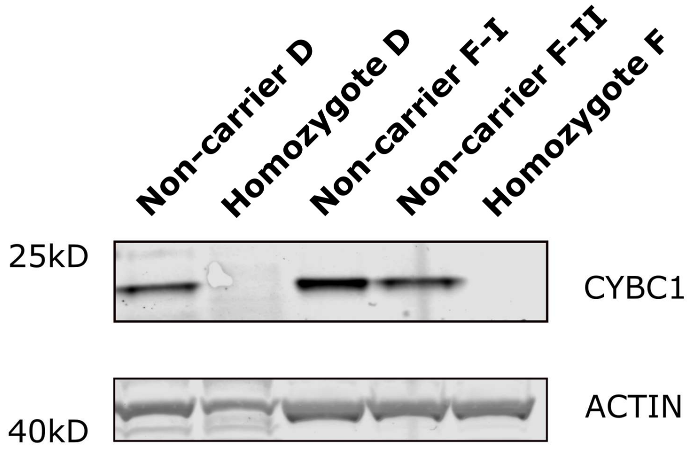

Supplementary Figure 3. CYBC1 protein expression in lymphocytes from *CYBC1* p.Tyr2Ter homozygous individuals D and F and their matched (age and sex) non-carriers. CYBC1 expression was not detected in the homozygous individuals in contrast to their matched non-carriers. The analysis was performed by western blot, and ACTIN was used as a loading control.

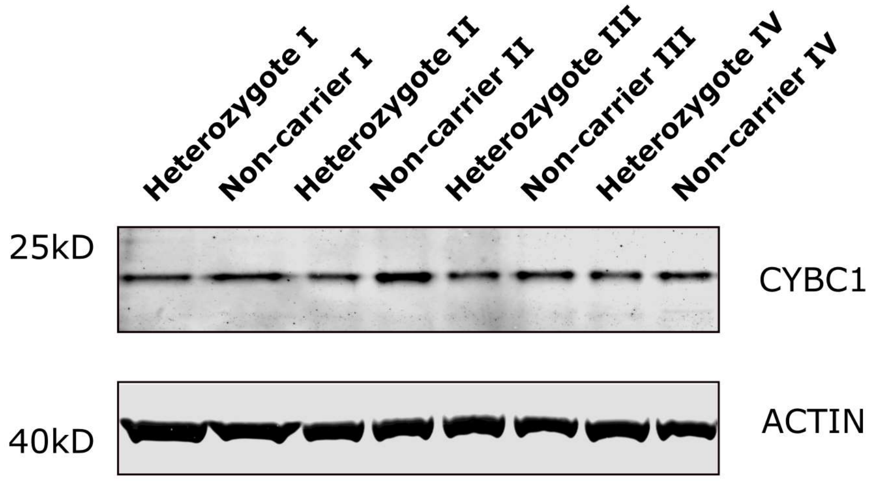

Supplementary Figure 4. CYBC1 protein expression in lymphocytes from four heterozygous carriers of *CYBC1* p.Tyr2Ter and a matched (age and sex) non-carrier for each. CYBC1 expression was reduced by approximately 53% in heterozygous carriers of p.Tyr2Ter compared to matched non-carriers. The analysis was performed by western blot, and ACTIN was used as a loading control.

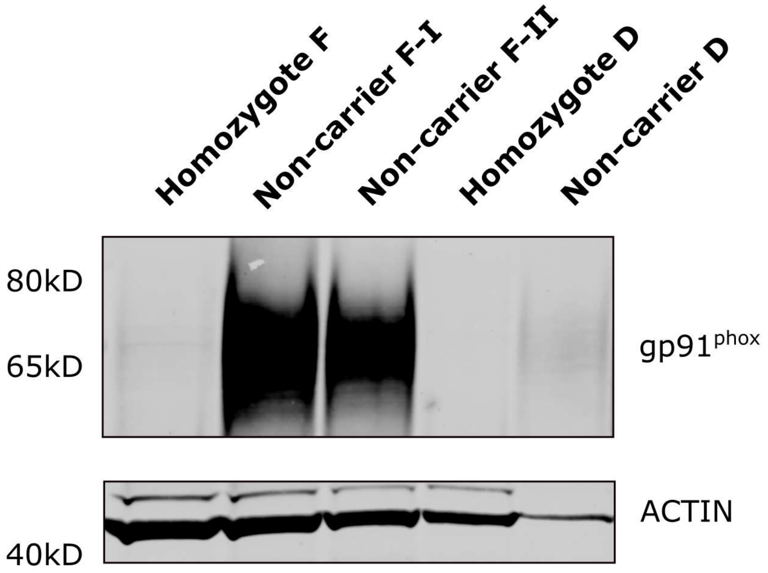

Supplementary Figure 5. gp91*phox* protein expression in monocyte-derived macrophages from *CYBC1* p.Tyr2Ter homozygotes and their matched (age and sex) non-carriers. Non-carriers F-I and F-II are matched for homozygote F, non-carrier D is matched for homozygote D. gp91phox expression was absent in the two homozygotes in contrast to their matched non-carriers. Non-carrier D had high expression of gp91*phox* relative to ACTIN (see quantification in Fig. 3d). The analysis was performed by western blot, and ACTIN was used as a loading control.

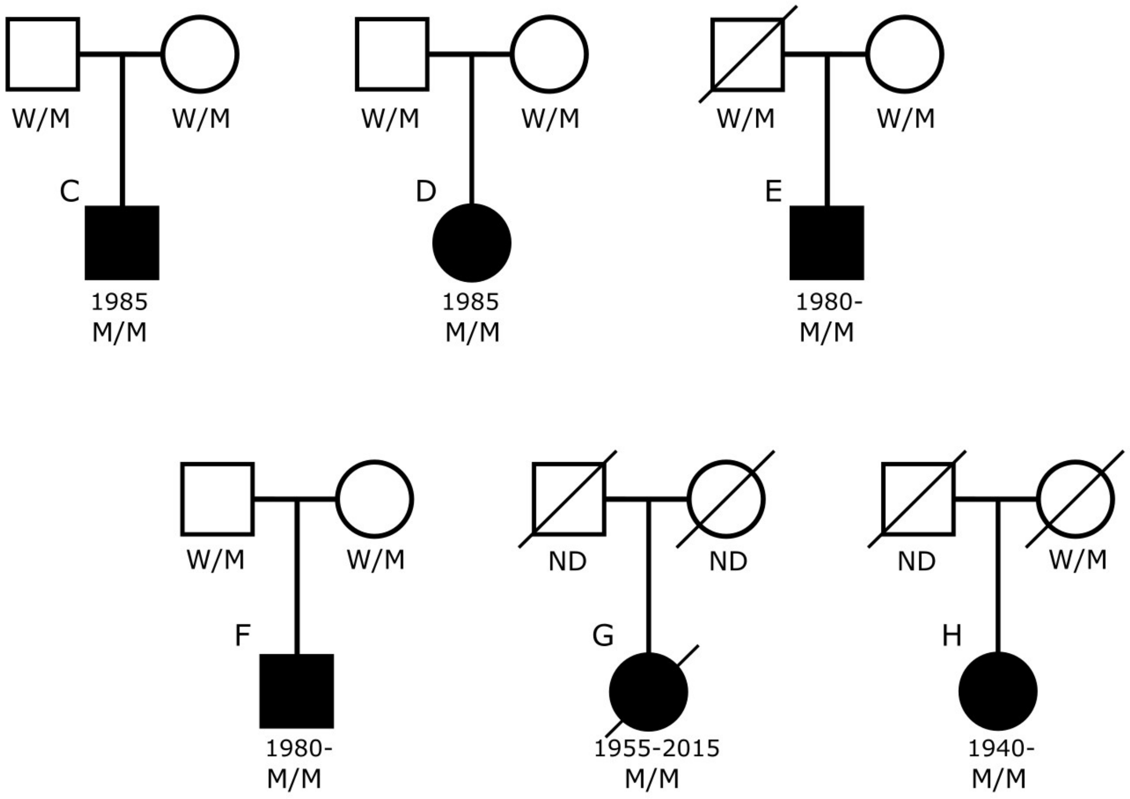

Supplementary Figure 6. Pedigrees of the six additional p.Tyr2Ter homozygotes (individuals C-H). Shown are the years of birth of the homozygous individuals (rounded to five years), and the genotype of *CYBC1* p.Tyr2Ter (NP_001028218.1:p.Tyr2Ter; NM_001033046.3:c.6C>G; hg38 position chr17:82,449,249). The genotypes are indicated with M and W, M representing the mutated allele and W the wild-type. M/M therefore indicates homozygous status, and W/M indicates heterozygous status, as determined by either WGS or chip-genotyping. ND indicates that the genotypic status could not be determined, true for three parents from whom DNA was not available for testing. Squares represent males, circles represent females, and slashed symbols indicate deceased individuals. Filled symbols represent affected individuals (referring to colitis, atypical infections, or an impaired PMA-induced neutrophil oxidative burst).

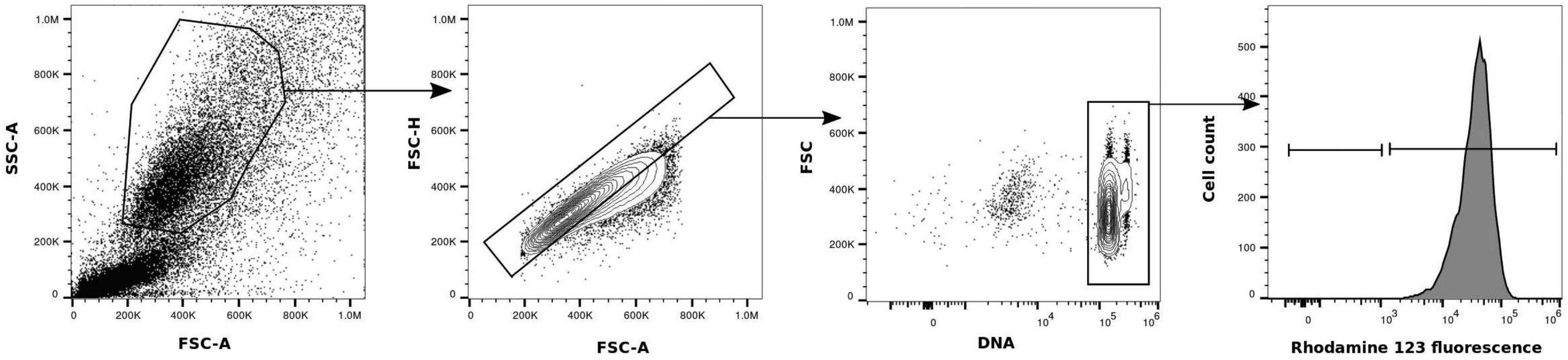

Supplementary Figure 7. Gating strategy for analysis of Rhodamine 123 fluorescence in neutrophils.

a

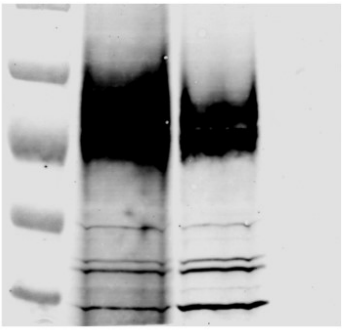

b

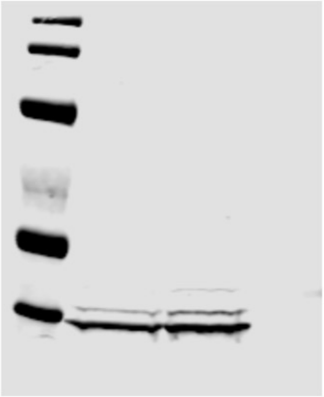

c

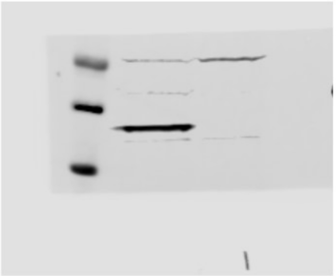

Supplementary Figure 8. Uncropped western blot accompanying Fig. 2d. Western blot of fresh neutrophils from *CYBC1* p.Tyr2Ter homozygous individual D (lane 3) and her matched (age and sex) non-carrier (lane 2). Lane 1 is a PageRuler protein marker. Membrane was physically cut in 2 parts, upper parts (a and b) for gp91^{phox} and β-actin detection, bottom part for CYBC1 detection (c). **a)** gp91^{phox} protein expression. **b)** beta-actin protein expression. **c)** CYBC1 protein expression.

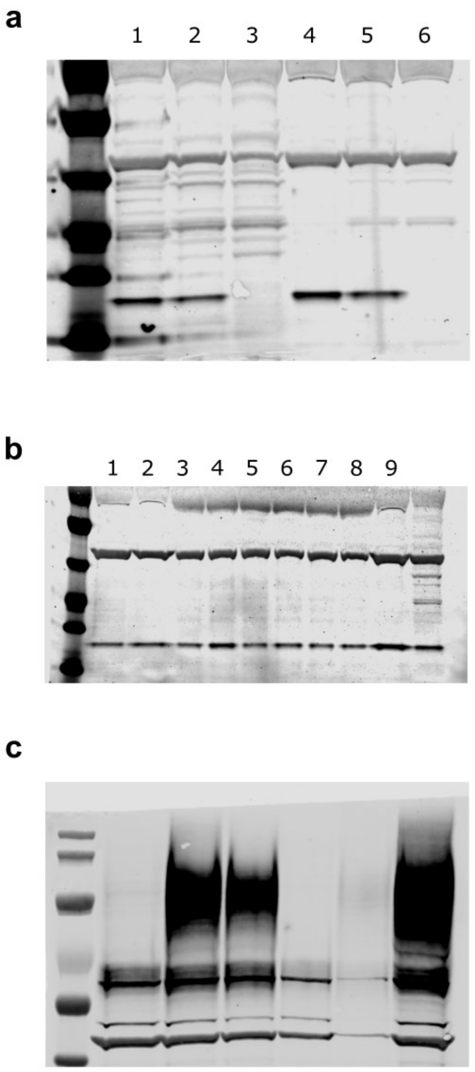

Supplementary Figure 9. Uncropped western blots accompanying Supplementary Figs. 3, 4, and 5. **a)** Western blot of lymphocytes from *CYBC1* p.Tyr2Ter homozygous individuals D and F (lanes 3 and 6) and their matched (age and sex) non-carriers, one matched for homozygous individual D (lane 2) and two for homozygous individual F (lanes 4 and 5). Membranes were stained with antibodies against CYBC1 (bottom band, approx 20 kDa) and β-actin (approx 42 kDa). Lane 1 is a B and T cell lysate from a previous experiment and serves as a control for the blot. **b)** Western blot of lymphocytes from four heterozygous carriers of *CYBC1* p.Tyr2Ter (lanes 1, 3, 5, and 7) and a matched (age and sex) non-carrier for each (lanes 2, 4, 6, and 8). Membranes were stained with antibodies against CYBC1 (bottom band, approx 20 kDa) and β-actin (approx 42 kDa). Lane 9 control did not have a matching heterozygous carrier. Lane 10 is a B and T cell lysate from a previous experiment and serves as a control for the blot. **c)** Western blot of monocyte derived macrophages from *CYBC1* p.Tyr2Ter homozygous individuals F and D (lanes 1 and 4), and their matched (age and sex) non-carriers, two matched for homozygous individual F (lanes 2 and 3) and one matched for homozygous individual D (lane 5). Membranes were stained with antibodies against gp91^{phox} (approx 100 kDa) and β-actin (approx 42 kDa). Lane 6 (not shown in Supplementary Fig. 5) is a monocyte derived macrophage lysate from a previous experiment and serves as a control for the blot.

Supplementary Tables

Supplementary Table 1. Average coverage of the six genes in which mutations are known to cause CGD, including *CYBC1*. Average coverage is given in A) our set of 37K WGS Icelanders, and B) in WGS data from each of the eight p.Tyr2Ter homozygous individuals.

**Gene**	**Average coverage in WGS set (×)**	**Average coverage for each individual (×)**							
		**A**	**B**	**C**	**D**	**E**	**F**	**G**	**H**
CYBC1	31,5	38,8	39,2	36,5	32,9	32,9	29,6	31,9	59,9
CYBA	30,1	32,9	39,6	35,2	36,6	31,2	28,5	34,5	59,5
CYBB	27,5	18,5	19,4	16,9	36,8	17,9	18,7	29,6	31,5
NCF1	16,4	41,8	39,0	37,5	33,9	33,8	28,7	33,3	52,5
NCF2	34,9	41,5	40,0	47,4	35,0	32,3	35,0	27,3	44,5
NCF4	32,4	38,7	38,0	40,5	31,6	34,0	33,1	27,2	49,2

Supplementary Table 2. Phenotypes of Icelandic individuals homozygous for NP_000092.2:p.Arg90Trp in *CYBA*.

**Gender**	**N offspring**	**Phenotype[1]**
Female	0	Chronic granulomatous disease
Female	1	Chronic granulomatous disease
Female	3	Sarcoidosis; Infections
Female	1	Sarcoidosis; Thyroiditis; Infections
Female	0	Death from septicaemia and hepatic failure

[1]Information based on hospital discharge diagnoses and death registry.

Supplementary Table 3. Association of *CYBC1* p.Tyr2Ter (rs778180128) with IBD and height in Iceland. The association is based on all chip-typed Icelanders and their close relatives (familial imputation) (N_{total}). The homozygous genotype frequencies are limited to chip-genotyped individuals (N_{chipt}). Test statistics are based on the likelihood ratio test.

**Trait**	**P-value**	**OR / Effect (95% CI)**	**Cases**			**Controls**		
			N_{total}	N_{chipt}	**Genotype frequency**	N_{total}	N_{chipt}	**Genotype frequency**
IBD	8.3×10^{-8}	67.6 (14.5, 15.5)	2,429	1,766	0.28% (1 in 353)	338,647	140,325	0.0014% (1 in 70K)
Height	3.3×10^{-4}	-1.24 SD (-1.92, -.56)	14,404	11,262	0.04% (1 in 2,252)	81,625	66,896	0.0030% (1 in 33K)

IBD = Inflammatory bowel disease; CI = Confidence interval; SD = Standard deviation.

Supplementary Table 4. Height information on the eight individuals homozygous for *CYBC1* p.Tyr2Ter. Height measurements were corrected for year of birth, sex, and age at measurement.

	Sex	YOB	Height in cm[1]	Height in SD[1]	Height percentile[2]
**Individual A**	M	1993	172	-0.81	9.6%
**Individual B**	M	1991	162	-1.88	0.4%
**Individual C**	M	1985	180	-0.24	32.6%
**Individual D**	F	1985	159	-0.99	6.0%
**Individual E**	M	1980	178	-0.24	32.5%
**Individual F**	M	1980	173	-0.73	11.9%
**Individual G**	F	1955	159	-0.78	10.5%
**Individual H**	F	1940	156	-1.12	4.4%

YOB = Year of birth; SD = Standard deviation.
[1]Average and SD for height of Icelandic males and females is 178.8±6.9 cm and 165.6±6.3 cm, respectively[4].
[2]We ranked 96,029 Icelanders for whom height measurements were available, from the shortest to the tallest. The height percentile refers to where, within these 96,029 individuals, each of the homozygous individuals rank.

Supplementary Table 5. Homozygous coding variants in the canonical transcript (NM_001033046.3 / NP_001028218.1) of *CYBC1* in Iceland[1].

**Position (hg38)**	chr17:82,449,249	chr17:82,446,679
**Reference allele**	G	G
**Alternative allele**	C	A
**MAF Iceland (%)**	0,76	1,50
**MAF gnomAD (%)**	absent	1,28
**Predicted impact**	high	moderate
**Gene**	*CYBC1 (C17orf62)*	*CYBC1 (C17orf62)*
**HGVSc**	NM_001033046.3:c.6C>G	NM_001033046.3:c.145C>T
**HGVSp**	NP_001028218.1:p.Tyr2Ter	NP_001028218.1:p.Leu49Phe

Supplementary References

1. Lek, M. *et al.* Analysis of protein-coding genetic variation in 60,706 humans. *Nature* **536,** 285–291 (2016).
2. Arnold, D. E. & Heimall, J. R. A Review of Chronic Granulomatous Disease. *Adv. Ther.* **34,** 2543–2557 (2017).
3. Gelling, R. W. *et al.* Lower blood glucose, hyperglucagonemia, and pancreatic α cell hyperplasia in glucagon receptor knockout mice. *Proc. Natl. Acad. Sci.* **100,** 1438–1443 (2003).
4. Benonisdottir, S. *et al.* Epigenetic and genetic components of height regulation. *Nat. Commun.* **7,** 13490 (2016).
